# Subsidized pharmacological treatment for smoking cessation by the Spanish public health system: A randomized, pragmatic, clinical trial by clusters

**DOI:** 10.18332/tid/111368

**Published:** 2019-09-05

**Authors:** César Minué-Lorenzo, Eduardo Olano-Espinosa, Isabel del Cura-González, Jose M. Vizcaíno-Sánchez, Francisco Camarelles-Guillem, José A. Granados-Garrido, Margarita Ruiz-Pacheco, M. Isabel Gámez-Cabero, F. Javier Martínez-Suberviola, Encarnación Serrano-Serrano

**Affiliations:** 1 Perales del Río Health Center, Dirección Asistencial Centro, Servicio Madrileño de Salud, Madrid, Spain; 2 Los Castillos Health Center, Dirección Asistencial Oeste, Servicio Madrileño de Salud, Madrid, Spain; 3 Area Medicina Preventiva y Salud Pública, Universidad Rey Juan Carlos, Madrid, Spain; 4 Unidad de Apoyo a la Investigación, Gerencia Asistencial de Atención Primaria, Servicio Madrileño de Salud, Madrid, Spain; 5 Red de Investigación Servicios de Salud en enfermedades crónicas, REDISSEC, Madrid, Spain; 6 Fuentelarreina Health Center, Dirección Asistencial Norte, Servicio Madrileño de Salud, Madrid, Spain; 7 Infanta Mercedes Health Center, Dirección Asistencial Norte, Servicio Madrileño de Salud, Madrid, Spain; 8 Guayaba Health Center, Dirección Asistencial Centro, Servicio Madrileño de Salud, Madrid, Spain; 9 Doctor Castroviejo Health Center, Dirección Asistencial Norte, Servicio Madrileño de Salud, Madrid, Spain; 10 Majadahonda Valle de la Oliva Health Center, Dirección Asistencial Noroeste, Servicio Madrileño de Salud, Madrid, Spain; 11 Los Fresnos Health Center, Dirección Asistencial Este, Servicio Madrileño de Salud, Madrid, Spain

**Keywords:** smoking cessation, drug therapy, primary health care, healthcare financing, Spain

## Abstract

**INTRODUCTION:**

Research has shown that financing drug therapy increases smoking abstinence rates, although most of these studies have been carried out in the private healthcare setting. The aim of this work is to assess the effect of subsidized pharmacological treatment on smoking cessation rates by the Spanish public healthcare system.

**METHODS:**

A pragmatic, randomized, clinical trial was performed by clusters. Randomization unit was the primary healthcare center and the analysis unit was the patient. Smokers consuming ≥10 cigarettes/day were randomly assigned to an intervention group that received financed pharmacological treatment or to a control group that followed usual care. The main outcome was self-reported or CO-confirmed continuous abstinence at 12 months. The main outcome, continuous abstinence rates (%), were compared between groups at 12 months post-intervention. A model was adjusted using mixed-effect logistic regression.

**RESULTS:**

A total of 1154 patients were included from 23 healthcare centers. In the intention-to-treat analysis, self-reported abstinence after 12 months in the control and intervention groups, respectively, was 9.6% (37/387) and 15.4% (118/767) (gender-adjusted OR=1.75; 95% CI: 1.1–2.8); for CO-confirmed abstinence the corresponding values were 3.1% (12/387) and 6.4% (49/767) (gender-adjusted OR=1.72; 95% CI: 0.7–4.0). Pharmacological treatment use was 35.1% (136/387) in the control group, and 58.3% (447/767) in the intervention group (adjusted OR=4.25; 95% CI: 1.8–9.9)

**CONCLUSIONS:**

Subsidizing pharmacological treatment for smoking cessation increases self-reported or CO-confirmed abstinence rates under realistic conditions in the primary care setting of the Spanish public health system.

## INTRODUCTION

An array of interventions have been shown to be both efficacious and cost-effective in helping patients quit smoking^1^. Among these are pharmacological treatments, which show a greater than twofold increase in the rate of successful cessation^1,2^.

Clinical practice guidelines recommend adding drug therapy to behavioral treatment of smokers attempting to quit, with some exceptions^1^. However, in the primary care setting, these types of interventions are uncommon, non-standardized^3–5^, and rarely include pharmacological treatment^5–7^. The treatment cost and lack of financing by the public healthcare system may be one of the reasons for not being more widely used^8^.

The Cochrane Systematic Reviews^9^ have assessed the effectiveness of funding treatments for smoking cessation and concluded that full financing of the treatment increases abstinence rates and number of attempts, almost doubling the rate of use of nicotine replacement therapy (NRT) and tripling that of Bupropion. Studies that compared the effect of funding the cessation obtained a combined relative risk for abstinence for at least 6 months of 1.77 (95% CI: 1.37–2.28) and 1.27 (95% CI: 1.02–1.59) in the case of full and partial funding, respectively.

A study has been published, using ecological momentary assessment methods with an interrupted time-lapse analysis, of fully financed smoking cessation treatments during 2011 by the Netherlands government, including drug therapy. In the studied period, primary care doctors prescribed more therapeutic drugs and smoking prevalence decreased. However, once the pharmacological coverage ceased, drug prescriptions decreased and smoking prevalence rebounded^10^. The number of smokers attended to by the Dutch telephone help-line for smoking cessation went from 848 in 2010 to 9091 in 2011 and dropped drastically after the funding ended in 2012 (only 151 in the first 18 weeks)^11^.

Pharmacological treatment is considered a complementary measure to others that decrease smoking addiction prevalence^11,12^ such as increasing tobacco taxes or smoke-free areas^13^. It is especially relevant for highly-dependent and low-income smokers, who show greater difficulties to quit smoking^14–16^, and has been associated with a decrease in hospital admissions due to cardiovascular disease^17^.

Most relevant studies have been conducted in the private healthcare setting, mainly in the USA^9^. In the public healthcare setting, two main studies have been performed, a pilot study with short-term outcomes in the United Kingdom primary care system^18^, whose National Health System is similar to the Spanish one, and a subsequent study in the Canadian Health System, which is public with private provision^19^. Primary care in Spain is organized in healthcare centers (HCCs), with public provision, where both health and non-health professionals work together to attend to users in their health area. Care for smokers, which is included in the services provided by health professionals as paid work, encompasses counselling, behavioral interventions, and suggesting pharmacological treatment at the patient’s expense. The aim of this study was to know whether funding pharmacological treatment for smoking cessation in primary care increases abstinence rates under realistic conditions.

## METHODS

A pragmatic, controlled, clinical trial was performed with paralleled groups randomized by clusters. The trial was approved by the Ethics Committee for Clinical Research of Hospital Doce de Octubre de Madrid.

Ninety-six HCCs in the Health System of the Community of Madrid were asked to participate. The HCCs were selected by convenience among those interested in participating. The inclusion criterion for HCCs was: having at least one doctor interested in participating in the trial. Twenty-nine HCCs, which provide health care to a population of 925000 citizens, aged >14 years, accepted to participate.

The inclusion criteria for the patients were: aged >18 years who attended the healthcare center for any reason between June and December 2009, smoking ≥10 cigarettes/day, at any stage of the smoking cessation process, attended by a general practitioner or nurse that addressed smoking cessation following usual clinical practice, and a signed informed consent. Pregnant and nursing women were excluded^1^.

### Sample size and sampling

Sample size was calculated considering that 8% of smokers treated with the intervention would stay abstinent after 1 year^20^, a clinical significance of 75% improvement post-intervention, a type I error of 5%, and a power of 80%. The obtained sample size was 459 patients. After controlling for the design effect, estimating an average cluster size of 25 patients and intraclass correlation coefficient of 0.02, and adding 20% of losses to follow-up, a total of 1632 smokers were included, with 816 per group. Allocation was performed by clusters, the primary HCC being the randomization unit. The 29 HCCs were assigned to the intervention or the control group following a simple, computer-generated random sequence (EPIDAT 3.1 software). Randomization was performed centrally by a researcher not involved in the study, and who was blind to the identity of the HCCs.

### Intervention

Behavioral treatment and recommendation for using pharmacological treatment were administered to both groups, in accordance with standard health services offered in primary care. In order to homogenize criteria and guarantee quality care for the smoker, general practitioners and nurses from the included healthcare centers received specific training as proposed by Olano et al.^21^.

Patients in the intervention group received first-line pharmacological treatment free-of-charge. The active ingredient (nicotine, Varenicline, or Bupropion) was chosen by the health professional in accordance with the patient’s preferences. Treatment doses were standard: NRT according to the number of cigarettes smoked, Bupropion at 150–300 mg/d and Varenicline at 1–2 mg/d. Treatment combinations were allowed at the discretion of the physician, such as NRT patch-plus or Bupropion and nicotine gum. The proposed standard duration was 8 weeks for NRT and Bupropion, and 12 weeks for Varenicline. Medication was distributed to the participating HCCs from the pharmacy services of the public health system. In the first visit, patients were given treatment for 2 weeks. At subsequent visits, they were given treatment for 1 month.

Patients in the control group were prescribed the treatment at the consultation and had to purchase it.

As it was a pragmatic trial, no visits other than standard practice were offered. Follow-up visits were left to the discretion of the professionals, according to their usual practice. The patients were followed for 12 months from the quit date, until December 2010.

Patients who did not intend to quit smoking were offered advice, but they were not given an appointment in either group.

### Variables

Main outcome variables were: continuous abstinence according to the criterion by Russel^22^ and biochemically validated abstinence through CO-oximetry. Appointments were given to patients that confirmed abstinence for validation via a CO-oximetry test, by Smokerlyzer Pico cooximeter, with a cut-off point of <7 ppm.

Secondary outcome variables were: use of pharmacological treatment and type of therapeutic drug (nicotine, Bupropion, and Varenicline). Data were collected at 12 months ±4 weeks from the quit date from electronic clinical records or, when information was not available, abstinence was confirmed via telephone call.

Baseline variables were: age, gender, educational level, socioeconomic level, daily cigarette consumption, pack-years, score in the Fagerström Test for Nicotine Dependence (FTND), previous attempts to quit, stage of change, and previous use of drug therapy.

### Statistical analysis

A descriptive analysis, of frequencies for qualitative variables and mean with standard deviation (SD) for quantitative variables, was performed. The groups were compared at the baseline in terms of outcome variables, descriptive variables, and prognosis factors. A bivariate analysis to compare variables was performed, using a chi-squared test and a Student’s t-test, in the case of dichotomous and continuous variables, respectively.

Main outcome was percentages of self-reported or CO-oximetry validated abstinence compared between the intervention and control groups, with 95% confidence intervals (95% CI). Intention-to-treat data analysis was performed at 12 months. Missing values for the main outcome variable were added using the ‘basal observation carried forward’ method. For the secondary aim analysis, effectiveness was evaluated by comparing the differences in the percentages of pharmacological treatment use at 12 months between both groups, and corresponding 95% CI.

A multilevel, logistic regression model was built at 12 months, where the dependent variable was self-reported (Yes/No) or CO-oximetry validated abstinence (Yes/No) or pharmacological treatment use (Yes/No), respectively, and the independent variable was the patient’s allocation group. Clinically significant variables were also tested as covariates, taking into consideration sampling by clusters.

Statistical SPSS 21 and STATA 14 software were employed for all calculations.

## RESULTS

**Figure 1:**
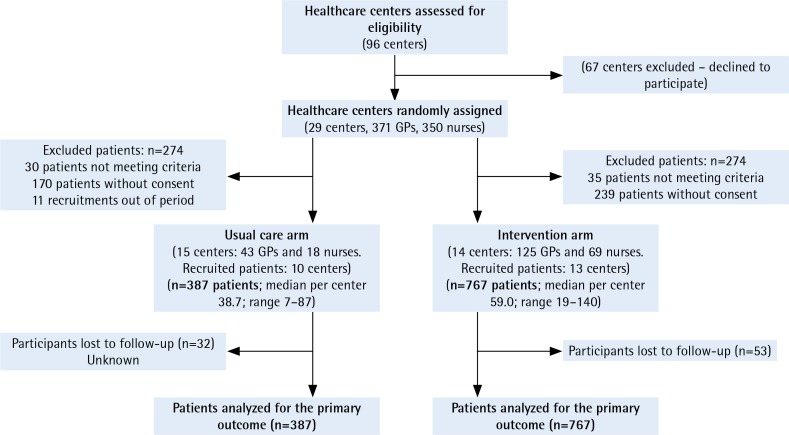
Flow chart of participants

A total of 255 health professionals from 23 healthcare centers participated in the trial and 1154 patients were included, 387 in the control group and 767 in the intervention group. Figure 1 shows the flow chart of participants throughout the trial. No differences were found in terms of number of consultations, rural or urban setting, and sociocultural level between the 6 non-participating centers (5 in the control arm and 1 in the intervention arm) and those participating in the study.

Baseline data were similar for both groups except that the intervention group comprised a larger percentage of men, smoked more cigarettes per day, and showed higher scores in the FTND (Table 1). Additionally, the rate of patients at the preparation and action stages of the cessation process was significantly higher in the intervention group.

**Table 1:** Baseline characteristics of participants by group

*Characteristics*	*Total n=1154*	*Control group n=387*	*Intervention group n=767*	*p*
**Age** (years), mean (SD)	46.04 (11.78)	46.6 (12.06)	45.8 (11.64)	0.268
**Gender**, n (%)				
Male	593 (51.4)	174 (45.0)	419 (54.7)	0.001
Female	560 (48.6)	213 (55.0)	347 (45.3)	
**Educational level**, n (%)				
No studies	25 (2.5)	4 (1.4)	20 (2.9)	0.184
Primary education	326 (32.9)	102 (34.7)	223 (32.4)	
Secondary education	387 (39.0)	105 (35.7)	279 (40.5)	
University	254 (25.6)	83 (28.2)	167 (24.2)	
**Annual income level**, n (%)				
<€26000	669 (69.7)	191 (65.6)	478 (71.4)	0.072
≥€26000	291 (30.3)	100 (34.4)	191 (28.6)	
**Cigarettes/day**, mean (SD)	22.0 (9.5)	20.6 (8.6)	22.6 (9.9)	0.012
**Years smoking**, mean (SD)	26.5 (11.9)	27.1 (12.4)	26.1 (11.6)	0.190
**Pack-years**, mean (SD)	29.6 (19.9)	28.6 (18.7)	30.2 (20.5)	0.220
**Number of previous attempts**, mean (SD)	2.3 (3.1)	2.2 (2.5)	2.4 (3.5)	0.159
**FTND**, mean (SD)*	5.4 (2.2)	5.2 (2.1)	5.5 (2.3)	0.035
**Previous drug therapy**, n (%)	178 (25.5)	68 (26.6)	110 (24.9)	0.625
**Stage of change**, n (%)				
Pre-contemplation	252 (22.2)	103 (26.6)	150 (19.6)	<0.01
Contemplation	239 (21.1)	105 (27.1)	134 (17.5)	<0.01
Preparation	385 (34.0)	113 (29.2)	272 (35.5)	0.03
Action	253 (22.3)	58 (15.0)	196 (25.5)	<0.01

* FTND: Fagerström test for Nicotine Dependence.

Losses to follow-up were similar in both groups, with values of 7% (n=54) and 8.5% (n=32) in the intervention and control groups, respectively (p=0.38). Compared to the intervention group, subjects lost to follow-up in the control group had a higher income level (70% vs 30%, p=0.045) and used less drugs (15% vs 85%, p=0.007).

With regard to the main outcome, the patients who self-reported having quit smoking at the 12 months follow-up were 118 (15.4%) and 37 (9.6%) in the intervention and control groups, respectively. Of these, 57 (48%) from the intervention group and 14 (38%) from the control group attended the CO-oximetry validation appointment, and abstinence was confirmed in the case of 49 patients (6.4%) from the funded-treatment group and 12 (3.1%) from the non-funded (Table 2). Abstinence rates were higher for subjects with higher income level for both arms (Table 3). The difference in abstinence rates and use of pharmacological treatment between high-income (>€26000/year) and low-income subjects (<€26000/year) was reduced after financing of the treatment (Table 3). Having used therapeutic drugs in previous attempts increased the success of quitting in the financed-treatment group (Table 4).

**Table 2:** Outcomes of continuous abstinence (self-reported or CO-validated) and use of pharmacological treatment, from mixed-effect logistic regression analysis

*Intention-to-treat*	*Control group*	*Intervention group*	*Rate difference*				

	*n (%)*	*n (%)*	*OR (95% CI)*	*OR raw (95% CI)^a^*	*p*	*AOR^b^ (95% CI)*	*p*
Self-reported continuous abstinence	37 (9.6)	118 (15.4)	5.8 (1.9–9.7)	1.84 (1.1–3.0)	0.01	1.75 (1.1–2.8)	0.02
CO-validated continuous abstinence	12 (3.1)	49 (6.4)	3.3 (0.8–5.7)	1.77 (0.8–4.1)	0.17	1.72 (0.7–4.0)	0.20
Pharmacological treatment use	136 (35.1)^c^	447 (58.3)^d^	23.1 (17.2–29.0)	4.52 (2.0–10.0)	0.00	4.25 (1.8–9.9)	0.00
NRT	46 (11.9)	131 (17.1)	5.2 (1.0–9.4)	1.80 (1.0–3.3)	0.05	1.77 (1.0–3.2)	0.06
Bupropion	45 (11.6)	135 (17.6)	6.0 (1.8–10.2)	1.58 (0.8–3.1)	0.19	1.49 (0.7–3.0)	0.83
Varenicline	49 (12.7)	199 (25.9)	13.2 (8.7–17.8)	3.40 (1.8–6.4)	0.00	3.41 (1.8–6.5)	0.00

a a Mixed-effect logistic regression

b AOR: adjusted odds ratio by gender

c 4 participants used Bupropion+NRT

d 10 used Bupropion+NRT; 7 Varenicline+NRT; 1 Bupropion+Varenicline.

**Table 3:** Outcomes of abstinence rates and use of pharmacological treatment by annual income level

*Group*		*Abstinence*		*Pharmacological treatment use*	

		*% (n)*	*OR (95% CI)*	*% (n)*	*OR (95% CI)*
Control group	<€26000	7.9 (15)		29.3 (56)	
	≥€26000	15.0 (15)	1.81 (1.0–3.3)	47.0 (47)	2.9 (2.0–4.2)
Intervention group	<€26000	13.4 (64)		54.8 (262)	
	≥€26000	19.4 (37)	1.4 (0.7–2.6)	62.3 (119)	1.9 (1.1–3.0)
Total	Usual practice	10.3 (30)		35.4 (103)	
	Financed treatment	15.1 (101)	1.5 (1.0–2.4)	57.0 (381)	2.4 (1.8–3.2)

**Table 4:** Outcomes of abstinence rates and use of pharmacological treatment by previous use of pharmacological treatment

*Group*		*Abstinence*		*Pharmacological treatment use*	

		*% (n)*	*OR (95% CI)*	*% (n)*	*OR (95% CI)*
**Usual practice**	No previous use of drugs	13.3 (25)		40.4 (76)	
	Previous use of drugs	10.3 (7)	1.2 (0.7–1.9)	51.5 (35)	2.3 (1.6–3.3)
**Financed treatment**	No previous use of drugs	15.1 (50)		60.5 (201)	
	Previous use of drugs	21.8 (24)	2.4 (1.0–6.0)	80.0 (88)	3.8 (1.9–7.3)
**Total**	Financed treatment	16.7 (74)		65.4 (289)	
	Non-financed treatment	12.5 (32)	1.4 (0.9–2.2)	43.4 (111)	2.5 (1.8–3.4)

For the secondary outcome use of pharmacological treatment was significantly greater in the case of the subsidized arm (58.3%) compared to the non-financed (35.1%) (Table 2).

Four per cent of the abstinence variability per year is explained by clusters (HCCs). The MOR (median odds ratio) between centers was 1.4, which can be interpreted as the increase in risk (median) that an individual would have if they were moved from one center to another with a higher risk.

All variables that showed basal differences and those that could impact the outcome from a clinical point of view were included in the multilevel model. After removing all non-significant variables, gender remained as the only adjustment factor. Self-reported continuous abstinence, adjusted by gender, was significantly greater in the intervention group (OR=1.75; 95% CI: 1.1–2.8); validated abstinence by CO-oximetry was also higher (OR=1.72; 95% CI: 0.74–4.0). Abstinence rates in both the intervention and control arms increased using NRT (OR=2.0; 95% CI: 1.3–3.0), Bupropion (OR=2.3; 95% CI: 1.5–3.4), and Varenicline (OR=3.0; 95% CI: 2.1–4.3) (Table 5). In the case of NRT, the impact was significantly greater in men (2.75; 95% CI: 1.6–4.6) than in women (1.2; 95% CI: 0.6–2.4).

**Table 5:** Outcomes of continuous abstinence (self-reported) with NRT, Bupropion and Varenicline

*Intention-to-treat*	*NRT use n (%)*	*No NRT n (%)*	*Bupropion use n (%)*	*No Bupropion n (%)*	*Varenicline use n (%)*	*No Varenicline n (%)*
Continuous abstinence	38 (21.5)	117 (12)	42 (23.3)	113 (11.6)	63 (25.4)	92 (10.2)
OR (95% CI)	2.0 (1.3–3.0)		2.3 (1.6–3.4)		3.0 (2.1–4.3)	

Success in quitting smoking was greater for men (OR=1.5; 95% CI: 1.1–2.2) and subjects with high-income level (OR=1.6; 95% CI: 1.1–2.3).

## DISCUSSION

Financing pharmacological treatment for smoking cessation in this trial significantly increased self-reported abstinence rates at the 12 months follow-up, and validated abstinence by CO-oximetry. The use of drug therapy also increased significantly, especially with nicotine and Varenicline. Additionally, the number of patients willing to attempt cessation in the intervention arm was much higher (Table 1).

A design by clusters was chosen considering the organization of the Spanish healthcare system and high risk of cross-contamination between participants. The trial was conducted in real-world conditions within the public health system context, with a high number of daily consultations, which forced patient recruitment to take place after randomizing the centers. Baseline differences shown in Table 1 appear to already indicate an intervention impact prior to its initiation. At the initial visit, patients were aware of the possibility of financing the treatment, which probably encouraged many participants to escape the first stages of the process and directly proceed to attempt to quit.

After refining the multilevel model, where gender remained as the only adjustment factor, differences in biochemically-validated abstinence lacked significance, which is probably due to the low ratio of participants who attended biochemical validation and the lack of power resulting from not reaching the calculated sample size.

This outcome is similar to other studies. Kaper et al.^23^ reported a biochemically-validated abstinence rate of 5.5% in the intervention group and 2.8% in the control group (OR=2.3; 95% CI: 1.2–4.1), and a self-reported abstinence of 7.8% and 5.5%, (OR=1.5; 95% CI: 0.9–2.4), respectively. Use of drug therapy was 10.8% in the financed group and 4.1% in the control (OR=2.9; 95% CI: 1.8–4.7).

Selby et al.^19^ conducted a pragmatic study with similar results after 6 months, and the Cochrane Review relative risk was within the same range^9^. A study by Twardella et al.^24^ with a similar design by clusters found a continuous abstinence rate of 9% for a group provided with training and financing compared to 1% success in a group treated following usual clinical practice.

Financing pharmacological treatment can be an important factor to help vulnerable populations quit smoking^14–16^. The higher the income, the greater the probability to cease smoking and the lower the tobacco consumption prevalence^25^. In this study, patients with a higher income level in both arms were more successful in quitting. However, in the intervention group, differences in drug use and abstinence rates were smaller between subjects with high- and low-income levels, which indicate a tendency to balance pharmacological treatment use as a result of funding it (Table 3). High income level was considered to be greater than €26000/year, the equivalent of more than 3 times the minimum interprofessional salary in the study period. The number of attempts to quit is considered a criterion of dependence: the greater the number of attempts, the greater the nicotine addiction. According to some studies^23^, smokers who have ever used drug therapy have more possibilities to re-use it if subsidized. In our study, smokers that had previously used drug therapy, used it more frequently, and also had greater chance to quit smoking (Table 4).

A study^26^ in the Netherlands reported the real-world experience of financing drug therapy in 2011 and compared the results with those from a 5% increase in tobacco prices at that time. Treatment funding yielded better health outcomes, although at a higher cost, but none of the mentioned strategies reduced the gap between social groups.

In most studies on this topic, NRT and Bupropion have been used. The latest also include Varenicline. All are considered first-line therapies in smoking cessation^1,2^. According to the latest reviews Varenicline is more effective than Bupropion and single-form NRT but not of the combination NRT^2,27^. In this trial, all drugs have shown their effectiveness in stopping smoking. The use of NRT and Bupropion doubles the chances of quitting smoking and Varenicline triples them. They are figures similar to those found in the literature^1^. Each one of the three drugs was more used when the patient did not have to pay for it. Therefore, NRT, Bupropion and Varenicline would be candidates to be subsidized. In Spain, the main experience of financing treatment took place in the Autonomous Community of Navarra, between 2003 and 2012. After 5 years, since December 2017, it is also subsidized in primary healthcare centers (HCCs), in the context of support and follow-up. In Madrid, a pilot funding experience was made in certain patients with chronic conditions for a few months in 2011. Treatments for other chronic conditions such as hypertension, diabetes, COPD, and alcohol abuse, have always been subsidized.

### Limitations and strengths

There are some limitations to this study. The funding requested for the conduct of the study was estimated considering that 15–20% of smokers would make an attempt to quit but the rate was much higher in both groups, likely due to the real offer to actually quit smoking^28^, together with a possible Hawthorne effect. Thus, the budget assigned to the project for funding pharmacological treatment ran out before completing the required sample size, since up to 58% of participants in the intervention group used pharmacological treatment. At the same time, the control group recruited less participants than expected, all of which resulted in a lack of power for some outcomes. This is probably related to the real-life impact of the intervention. Primary healthcare interventions are scarce^3–8^. The possibility of giving a treatment leads to an increased intervention by the practitioner, so many participants decided to make an attempt. In contrast, in the control group, recruitment was slower in the same period since interventions are rarer.

It is not to be expected that financing a drug will increase its efficacy, but that the number of attempts to quit smoking and the use of pharmacological support will be increased. Professionals attract more patients because they offer more financed treatment and patients decide to make an attempt as they know that treatment will be funded. The main reason for the difference in the distribution of the stages of change is most likely the treatment offer itself, which is the subject of the study, although selection bias cannot be discarded. This is why the multilevel analysis was not adjusted for the stages of change.

The calculation of the number of patients who would attempt to stop smoking was done according to the theoretical framework of the stages of change. Some authors suggest a different theoretical framework, called the ‘Catastrophic theory’^29^, which considers motivation as a dynamic, fluctuating process where diverse stimuli can trigger attempts to quit. Even small triggers can lead to sudden ‘catastrophic’ changes. One of those stimuli that could trigger quit attempts could be the immediate offer of a funded treatment. In a clinical trial by Jardin et al.^30^, both motivated and unmotivated patients were given free NRT and referred to a telephone help-line for cessation, and similar abstinence rates were observed. Funding the treatment can be one trigger stimulus for less motivated smokers to attempt cessation. This may explain differences between groups across the different stages of the change process, and consequently in the number of quit attempts.

As is the case for studies by clusters, another possible limitation is the selection and attrition biases. The control arm recruited fewer patients, possibly due to the lack of involvement of health professionals not assigned to the intervention arm, and a higher number of centers in the control arm did not recruit any patients. Another bias can be the temporariness of the funding assigned to the project, of which health professionals were aware of and which may have influenced their decision to recruit more patients willing to quit smoking for the intervention group^28^. Identification of patients prior to randomization or blinding of professionals in charge or recruitment was not possible.

Another limitation is the irregular distribution of HCCs (cluster) in the two arms. The number of professionals in the control group (doctors and nurses) was much lower than in the intervention group (Figure 1), which may have influenced the lower uptake of patients in the control group. Despite this, the average uptake per center was not much lower in the control HCCs.

Among the strengths of this study is its pragmatic design^31^ for assessing the effect of treatment funding under real-life conditions. Recruitment, training of health professionals^21^, information and assistance for patients, and characteristics of healthcare centers and professionals were identical in both groups. Criteria for inclusion, flexibility to apply the intervention, comparison with usual practice, absence of formal, controlled follow-up visit, and the intention-to-treat analysis were also part of our pragmatic design. Smokers were treated by their assigned health professionals at usual consultations, without further limitations. Situations where pharmacological treatment is not indicated was the only exclusion criterion^1^. We consider that these results can be applied to real-world primary care consultations in the Spanish setting. Drug financing has not been modified in our environment since the trial was conducted.

An intervention on smoking dependence does not consist of a mere pharmacological prescription. Treatment mainly consists of behavioral counselling. However, use of drug therapy is an important facilitator, especially in the case of underprivileged patients, as is the case for the highly-dependent or those from a low socioeconomic level. Hence, funding this treatment in a manner similar to other treatments already financed by the public system is a key factor.

## CONCLUSIONS

Financing pharmacological treatment of smoking addiction by the public health system increases abstinence rates and offers the opportunity to improve interventions for tobacco consumption in primary care, with increased number of attempts to quit and use of therapeutic drugs.
