# Supplementary material for: Subsidized pharmacological treatment for smoking cessation by the Spanish public health system: A randomized, pragmatic, clinical trial by clusters
Source: Tob Induc Dis. 2019 Sep 5;17:64. doi: 10.18332/tid/111368 (PMC6770612; doi:10.18332/tid/111368)
Supplement: Supplementary file 1 [file TID-17-64-s1.pdf]

ACKNOWLEDGEMENT OF PERSONNEL OF PARTICIPATING HEALTH CENTERS

Grupo FTFT-AP

Algete Health Center: Victoria Aguirre-Trigo, Rosa de Lima López-Pérez, Montserrat Blas-Escribano, Mª Dolores Mugarza-Hernández, Gloria Sánchez-López, Ángeles Díaz-Entresotos-Cortés, Aida Fátima López-Laguna, José Manuel Valverde-Bachiller, José Aurelio Formento-Tirado, Juan Antonio Ortega-Córdoba, Silvia Rodríguez.

Aranjuez Health Center: Clara Ochoa-Ruiz, Luis Basurto-Ruiz, Mª del Mar Martín-Ruiz, Juan A. Ortiz-Pallarés, Mónica López-Rodríguez, Luis Santiago Mesa-Santos, Ángel García-Parrilla, Pedro Samblas-Tilve, Manuel Navas-Alonso.

Aravaca Health Center: Mónica Nácher-Conches, Mercedes Colomo-Rodríguez, Eva Moreno.

Barrio del Pilar Health Center: Mª Ángeles Nieto-Díez, Mª Ángeles Brieva-García, Mª Patrocinio Verde-González, Mª José García-Sánchez, Azucena Sáez-Berlana, Victoria Cantera-Urcía, Esther Labrador-Arranz, Pilar Martín-Cerrato, Ángel Castellanos-Rodríguez, Ana Román.

Ciempozuelos Health Center: Mª Cruz León.

El Restón Health Center: Nuria de la Peña-Antón.

Fuentelarreina Health Center: Mª Felisa Núñez-Sáez, Mª Concepción Díaz-Laso, Rafael Ruiz-Morote, Orlando Enríquez-Dueñas, Mª Luisa Asensio-Ruiz, Cristina Gracia-Bouthelier, Victoria Díaz-Puente.

Galapagar Health Center: Beatriz Fernández-Navarro, Paloma Henares-García, Mª Nieves Puente-García, Concepción Marcelló-Andrés, Macarena Espejo-Sauceda, Cristina Prieto-Granado, Elena Villa-Fernández.

Guayaba Health Center: Carmen Gil-Vela, Elena Martínez-Salinero, Priscila Muñoz-San Juan, Karima Elaachir-Núñez, Cristina Sánchez-García, Mauricio Sainz de la Maza-Aparicio, Concepción Vargas-Machuca, Irama Valerio-Viloria, Victoriano Fraga-Canora, Bárbara Bregón-García, Mª Ángeles Gómez-Medina, Ignacio Bordas-Rodríguez, Sonia Sánchez-Yubero, Sonia Álvarez-Mesuro, Carmen Hernández-Manzano, Mª Dolores Peña-Díaz, Elia Arranz-Martín, JMª Ferrer García-Borrás, Alejandra Bravo-Pereira, Andrea Ramos-Castillo, Mª Luisa Fernández-Tola.

Infanta Mercedes Health Center: Fernando López-Pañamil, Javier Castellanos-Martínez, Pilar Cabezón-Blanco, Esther Moreno-González, María José García-García, Concha Seco-Del Palacio, José Gómez-Fernández, Concha Niño-Aguado, Mercedes Martinez-Acevedo, Carmela Salvador-Armendáriz, Elena Fernández-Pérez, Beatriz Cáceres-Sánchez, Carmen Resino-González, Ana Balboa-Ruiz, Inmaculada Cuevas-López, Máxima Miguel-Benedito, Wael Nasrallah-Kauzam, Leticia Guerrero-Galindo, Dolores Rodero-Fons, María Rodríguez-Portillo.

Legazpi Health Center: Mª Teresa Recio-García, Carlos Martínez-García-Olalla, Urbano García-Campos, Carlos Casado-Álvaro, Marta Bosom-Velasco, Amparo Díez-Canseco, Isabel Pérez-Soria, José Francisco Albertos-Campos, Margarita Gutiérrez-Salas, Carmen Macías, Cristina Ibáñez, Cristina Reales, Montserrat Sebastián, Emilio Sedeño, Mercedes Fuentes, Juan Antonio Bravo, Magdalena Rodríguez, Elena González, José Manuel Carreño.

Los Rosales Health Center: María José Piñero-Acín, Juan Vicente-Ruiz, Julio Sagredo-Pérez, Carmen Terrón-Rodas, Rafael Gómez-Moreno, Magdalena Undabeitia-Pérez de Mezquia, Mª Concepción Prieto-García-Calderón, Carmen Sánchez-Otero, Inmaculada Antequera-Medina, José Ramón Hernández-Pardo, Francisco Javier Zufía-García, Belén Navarro-Carnero, Mª José López-Gómez, Isabel Calvo-García, Rocío Horcajada-Alocén, Isabel Muñoz-Quesada, Rosa Ana García-Pérez, Mª Victoria Gamo-González, Laura Laina-Laina, Pilar Sanchez-Aguilera, Mª Luz Collado-Arribas, Cristina Calero-Tomás, Nuria García-Alonso, Carmen Gómez-Ortiz, Alberto Cano-Pérez, María Suárez-Baraza, Amparo García-Romanos.

Majadahonda Valle de la Oliva Health Center: Cristina Sosa-Díaz-Saavedra, Carmen Fenoll-Brotons, Pilar Nieto-Sánchez, Esther Minguela-Puras, José Manuel Blanco-Canseco, María Justicia-López, Mónica Gracia-San Román, Mª Dolores Castaño-Jover, Paloma Gómez-Sanz, Mª Jesus Salvador-Morán, Carmen Cuchillero-López, Rubí Sobrino-González, Juana María Gómez-Puente.

Manzanares Health Center: Armando Varela-González, Esperanza Calvo-García, Magdalena Coca-Díaz, Lucía Sierra-Santos, Alberto González-Hernández, Dori Clemente-Fernández, Jesús Castro-Toro, Verónica Gómez-Garrido y Mª Jesús Antolín-San Martín.

Mirasierra Health Center: Manuel Gómez-García, Encarnación Pérez-Pérez, Alicia Muñoz-Núñez, Jesús Zambrano-Álvarez, Joaquín Morera-Montes, Mª José Lucena-Martín, Isabel Elena-Gándara.

Monterrozas Health Center: Asunción Fernández-Pascual.

Orcasitas Health Center: Virginia Lasso, Olga Martín, David Agüero, Cristina Gutiérrez, Susana Duce, Pilar Moreno-Cano, Lucía Fernández, Aurora Fernández, Isabel Estrada, Javier Martín, Almudena Hormigos, Teresa Esteban, Esther Luna.

Paseo Imperial Health Center: Isabel Sánchez-Barba, José Antonio Fernández-Ruiz, Mª Rosa Hernández-Racionero, Andrés J Caballero-Gallego, Mª José Alonso-Marcos, José Pérez-Rodríguez, Elena Collada de la Fuente, María Carrera-Lavín, Jorge Olmedo-Galindo, Isabel Ramos-Aldavero, Rocío de Vicente-García-Ochoa, Ana Elena Hoyo-Fernández, Yolanda Hernández-Hernández, Mª Isabel Molero-Fernández de Sevilla, Carmen Sáenz-Rodríguez, Amaya Paniagua-Díaz, Eva Serradilla-Fernández, Mª Ángeles Garro-Palancar, Verónica Toril-Molina, Raúl Arroyo-Álamo, Carmen Fernández-Box, Rosario Periñán-Orellana, Dolores Roca-Cañizares.

Perales del Río Health Center: Mª Ángeles Usero-Martín, Mercedes Rojo-Tardón, Isabel Sánchez-Fonseca, Patricia Magallón, Eloína Delgado-Silván.

Pozuelo San Juan Health Center: Pedro Pablo Iglesias-Dorado, Fernando León-Vázquez, Mª Pilar Burón-Martínez, Alicia Díaz-Revilla, Concepción Fouz-López, Adela Rodríguez-Martín, Manuel Bernardo-Corral, Jesús Ignacio González-Orodea, Mª Dolores Méndez de

Pando, Luis García-Hernández, Mª José Rey-Álvarez, Lourdes Álvarez-González, Mª Luisa Cea-Jiménez, Mª Rosario Febrel-Torcal.

Rosa Luxemburgo Health Center: Álvaro Undabeytia-Cilla, Teresa Martínez-Arija, Francisco José Salcedo-González, Mª Luisa Galán-Leal, Javier Rodríguez de Frutos, José Luis Segovia de Pablo, Asunción Garrido-Garrido.

Valdemoro Health Center: Javier Huerta-Dante, Mª Luisa Fernández, Rosario Morales, Beatriz Remón, Mª Jesús del Villar, Rafael Rodríguez-Cabanillas, Mª Angeles Morales-Gómez, Belinda Añover-Rodríguez, Marta Corbacho-Martín y Rafael Verdugo-Martín.

Villanueva de la Cañada Health Center: Jon Koldo Sagardui-Villamor, Mireia Rey-Pérez de Pipaón.

Servicio Farmacia Atención Primaria Servicio Madrileño de Salud: Almudena Añino-Alba, Lucía Jamart-Sánchez, Ascensión Pérez-Redondo, Ana Domínguez-Castro.

© 2019 Minué-Lorenzo C.
